# Estimation of habitual intake of infrequently consumed nutrients using the mixture distribution method

**DOI:** 10.3389/fnut.2025.1631495

**Published:** 2025-11-10

**Authors:** Smitha Joseph, Santu Ghosh, Sumathi Swaminathan, Tinku Thomas

**Affiliations:** 1 Research Scholar, Manipal Academy of Higher Education (MAHE), Manipal, India; 2 Division of Epidemiology and Biostatistics, St. John's Research Institute, St. John's National Academy of Health Sciences (SJNAHS), Bangalore, India; 3 Department of Biostatistics, St. John's Medical College, and Hospital, SJNAHS, Bangalore, India; 4 Division of Nutrition, St. John's Research Institute, SJNAHS, Bangalore, India

**Keywords:** habitual intake, measurement error, 24-h recall, gamma regression, beta-binomial distribution

## Abstract

**Background:**

The habitual intake of infrequently consumed nutrients typically exhibits a highly skewed distribution, primarily driven by the reported consumption and non-consumption of nutrients in repeated 24-h dietary recalls. The current methods for estimating this distribution are often computationally intense.

**Methods:**

A mixture distribution method (MDM) was proposed to estimate habitual intake distribution of infrequently consumed nutrients, in which the frequency of consumption of a nutrient was modeled using a beta-binomial distribution and the amount consumed using a gamma distribution. The habitual intake using this method was compared to the Iowa State University Foods (ISUF) method using sample data consisting of four non-consecutive 24-h diet recalls collected from 120 children aged 6–59 months in Bihar, India. To assess the impact of zero inflation on the estimation of habitual intake, nutrient intakes were simulated with varying percentages of positive intakes, and habitual intakes were calculated using both methods.

**Results:**

The median (IQR) habitual intakes estimated from the MDM and ISUF methods were 0.47 mg (0.29, 0.65) and 0.46 mg (0.29, 0.62) for vitamin B_6_ and 0.38 mcg (0.14, 0.68) and 0.40 mcg (0.18, 0.69) for vitamin B_12_, respectively. Similarly, comparable results were found for other nutrients such as vitamins B_3_, B_5_, B_12_, and A and iodine. The simulated data showed that the estimated habitual intake by the MDM increased with the proportion of positive intakes considering the higher probability of consumption. When the proportion of positive intakes was below 60%, the estimates using the MDM, which considers the probability of consumption, were higher than the arithmetic mean calculated from 15 recalls.

**Discussion:**

The proposed MDM offers a computationally simpler approach to estimate habitual intake distribution by modeling the probability distribution of non-consumption and the distribution of positive intakes. The procedure can be easily implemented using standard statistical software and estimates habitual intake for infrequently consumed nutrients from multiple 24-h dietary recalls.

## 1 Introduction

Accurate dietary assessment plays a crucial role in public health by providing the evidence base needed to design, implement, and evaluate nutrition-related policies and interventions. Reliable information on dietary intake helps identify nutrient deficiencies, excesses, and dietary patterns associated with chronic diseases such as obesity, diabetes, and cardiovascular disorders. It also supports the monitoring of population-level dietary trends, enabling timely action to address emerging nutritional challenges. In public health practice, well-conducted dietary assessment informs food fortification programs, dietary guidelines, and health promotion strategies tailored to specific population groups. Ultimately, improving the precision of dietary assessment enhances the effectiveness of nutrition policies and contributes to better health outcomes at the population level.

The public health policies and nutritional recommendations are based on the relationship between long-term nutrient intake and health outcomes but not short-term consumption. The usual dietary intake, or habitual intake, provides the average amount of food or nutrient consumed by an individual over a long period (1). The accurate estimation of habitual intake at the population level is crucial for understanding the diet–health relationships and variability of food and nutrient intake, which requires multiple 24-h dietary recalls (24HR). However, the food and nutrients consumed on a fraction of the sample days of recall for a portion of the sample are considered infrequently consumed (2). Therefore, nutrients that are not consumed daily—such as vitamins B_12_ and E—can be considered as infrequently consumed nutrients (3). Capturing the intake of these nutrients requires a greater number of recalls to differentiate between true consumers and non-consumers (4).

The measurement error model used for habitual intake estimation assumes an approximately symmetric intake distribution (5). While commonly consumed nutrients can be transformed to meet this assumption by simple power or log transformation, infrequently consumed nutrients are typically positively skewed (6, 7). These nutrients present additional challenges, including a high proportion of non-consumption during recall and a skewed intake among consumers. The variability is also influenced by age, sex, ethnicity, and seasonality (7–9).

The Iowa State University Foods (ISUF) method is used to estimate the habitual intake distribution of infrequently consumed foods and nutrients. It uses a two-part model with person-specific effects: the first part models the probability of consuming a certain food or nutrient using a mixture of binomial probabilities and the second part models the intake amount of the food or nutrient when consumed (2). This approach modified the measurement error model to account for the mixture of the consumers' and non-consumers' intake distributions. Similar methods including the National Cancer Institute (NCI) method (7), the Statistical Program to Assess Dietary Exposure (SPADE) method (10), and the Multiple Source Method (MSM) (11) differ in their two-part model implementation of estimating frequency and the amount of consumption.

However, to use these methods, the intake data must be appropriately transformed to align with the measurement error model to estimate habitual intake. The second part of the ISUF method involves a two-step transformation based on the Iowa State University (ISU) method to handle highly skewed intake distributions. Together, these steps make estimating habitual intake for infrequently consumed nutrients a complex process.

This study proposes a computationally simpler approach built on the mixture model framework of the ISUF method to estimate the habitual intake distributions for infrequently consumed nutrients.

## 2 Methods

Nutrients consumed on fewer than 90%−95% of the recorded days were classified as infrequently consumed nutrients (4). To assess the intake distribution, a histogram was used to distinguish between regularly and infrequently consumed nutrients. For infrequently consumed nutrients, a substantial portion of the sample reported no intake during recall days.

### 2.1 Estimation of habitual intake for infrequently consumed nutrients

The habitual intake distribution of infrequently consumed nutrients consists of zero-inflated data from non-consumers and a skewed intake data from consumers on recall days. The ISUF method assumes that the habitual intake of an individual on consumption days is independent of the probability of consumption of the nutrient under study. Thus, habitual intake of the nutrient on all days of recall can be modeled as the individual's habitual amount of intake on consumption days (the conditional distribution of positive intakes) multiplied by the individual's probability of consuming the nutrient on any recall day (2).

Let *Y*_*ij*_ be the observed intake for individual i on day j of recall, *y*_*i*_ represent the habitual intake of individual i, and *p*_*i*_ be the probability that an individual i consumes the nutrient on any given day. Let $Y_{ij}^{*}$ be the observed positive intake and $y_{i}^{*}$ be the corresponding habitual intake. Then, the ISUF model is


$$y_{i}=y_{i}*ip_{i}\;;p_{i}\sim D\left(p;\theta \right)$$ (1)

where *D(.)* is a suitable probability distribution of *p*_*i*_. In the ISUF method, the habitual intake of the amount consumed on consumption days was estimated using the ISU method as explained by Nusser et al. (6) which requires a two-step transformation of nutrient intake to normal distribution. The consumption probability distribution was modeled as a discrete set of equally spaced probabilities (ranging from 0.0 to 1.0), with specific probability masses for the number of days of recalls. The proportion of individuals consuming the nutrient on *l* out of *r* days (where *r* is the number of days of recalls and *l* ranges from 0 to *r*) was derived from the combination of those binomial probabilities with the weighted probability mass estimated using the modified minimum chi-square estimator (2, 12).

### 2.2 Mixture distribution method (MDM) of infrequently consumed nutrients

This study suggests two modifications to the ISUF method. First, the conditional distribution of habitual intake on consumption days, ${y_{i}}^{*}=E\left(Y_{ij}|i,Y_{ij}>0\right)$, is modeled using a gamma distribution to account for skewness in observed intake. Second, the distribution of probability of consumption is estimated by modeling the proportion of consumption days from multiple recalls by beta-binomial probability distribution (13) to account for potential overdispersion and varying probability of consumption.

The practical application of gamma distribution to model positive intakes have been evaluated against lognormal and mixture normal distribution by comparing their Akaike information criteria (AIC) values. The data on frequency of consumption were examined for the best-fitting distribution among binomial, Poisson, negative binomial, and beta-binomial distributions. The AIC values are given in Supplementary Table 2. The merit of gamma distribution for modeling skewed nutrient intake is explained elsewhere (14, 15).

As an alternative to transforming individual non-zero or positive nutrient intake data to normal variate, we modeled it using non-normal distribution specifically gamma probability distribution. If *Y*_*ij*_ is distributed as gamma with pdf:


$$\begin{array}{l} f_{y}\left(y\right)=\;\frac{\text{λ}}{\;\Gamma \left(\nu \right)}{\left(\text{λ}y\right)}^{v-1}e^{-\text{λ}y},\;y>0,\;v>0,\text{ λ}>0, \end{array}$$ (2)

where mean, $E\left(Y\right)=\frac{v}{\text{λ}},\;and\;Var\left(Y\right)=\;\frac{v}{{\text{λ}}^{2}}=\frac{mean^{2}}{v}$. Here, Γ(ν) was the gamma function, and λ and *v* were scale and shape parameters, respectively.

Let ${{\text{Y}}_{\text{i}\text{j}}}^{*}$:{*i* = 1, 2, ...., *n, j* = 1, 2, ... , *r*_*i*_} denote the set of unadjusted positive observed intakes for a dietary nutrient, where n is the number of individuals with at least one positive intake and *r*_*i*_ is the number of positive intake days for individual *i*.

The unobserved positive habitual intakes were modeled using gamma distribution with a log link within a measurement error framework as follows:


$$\log\left(E\left\{Y_{ij}*\right\}\right)=y_{i}*+u_{ij}$$ (3)

where ${{\text{Y}}_{\text{i}}}^{*}$ was the unobserved positive intake of individual *i* with mean μ_y_ and variance $\sigma_{\text{y}}^{2}$, and **u**_**ij**_ was the unobserved measurement error with mean 0 and variance $\sigma_{\text{u}}^{2}$. The variance $\sigma_{\text{u}}^{2}$ represented within-individual variance, and $\sigma_{\text{y}}^{2}$ represented the between-individual variance in intake or the variance of habitual intakes.

The estimates of {μ_*y*_, σ_*y*_, σ_*u*_} were obtained by the gamma random effect model, and the habitual positive intake was obtained as follows:


$${\hat{z}}_{i}=\log\{\;{\hat{y}}_{i}\}=\hat{\alpha }+\frac{{\hat{\sigma }}_{y}}{\sqrt{{\hat{\sigma }}_{y}^{2}+\frac{{\hat{\sigma }}_{u}^{2}}{r}}}(z_{i}-\hat{\alpha })$$ (4)

where $z_{i}=\log\left(y_{i}\right),\;\log\left({\hat{\mu }}_{y}\right)=\hat{\alpha }$, intercept of gamma random effects model, and ${\hat{\sigma }}_{y}$ the estimate of between-individual variability and ${\hat{\sigma }}_{u}$ the estimate of within-individual variability. Finally, ŷ_*i*_ could be estimated by exp (ẑ_*i*_).

The probability of the positive intake (*p*_*i*_) was estimated by the beta-binomial probability distribution fitted to the frequency of positive intake for r repeated 24-h recalls. The maximum likelihood estimation technique was used to estimate the parameters of the distribution.

Thus, habitual intake was obtained by the Equation 1 as follows:


$$\begin{array}{l} y_{i}^{*}={ŷ}_{i}\times {\hat{p}}_{i}\;\forall \;i=1\ldots n \end{array}$$ (5)

Both regression methods—gamma regression and beta-binomial regression—can easily be implemented in standard statistical software. R package “lme4” was used for the calculation of within- and between-individual variability for the estimation of habitual positive intakes using the gamma regression method. A package named “VGAM” was used for the estimation of probability of consumption using beta-binomial distribution. The R-program code for executing the MDM method is provided in Supplementary material 1.

### 2.3 Data used for application of the methods

Two surveys were conducted in a cohort of households in Gaya and Nalanda districts of Bihar state during two seasons—the first season between July and August 2019 and the second season between December 2019 and January 2020, to examine the production, distribution, and consumption of nutrient-rich foods. The primary variable of interest was the anthropometric growth of children aged 6 to < 60 months. Two 24-h dietary recalls, including breastmilk intake, were performed for children aged 6 to < 60 months in the sampled households during the two seasons. The details of the sampling procedure, sample size, and other nitty-gritty of this study are described elsewhere (16). A sample of 120 participants with intake data on all four recalls available (Supplementary Figure 1) was considered for the current analysis. The trained interviewers conducted face-to-face interviews with mothers to collect the 24-h dietary recalls from their children. The second recall was captured on a non-consecutive day. First, the participant listed the foods and beverages consumed during the previous day, including vitamin and mineral supplements from when the child woke up, and for the next 24 h, using food portion size aids (utensils commonly used by the community to eat food). Following this, interviewers assisting in their recall asked queries about breastfeeding habits and foods that they may have forgotten to report, such as snacks, foods consumed during special occasions, and the timing of food consumption. Nutrient data were analyzed using an MS Excel calculator created using the food composition database developed specifically for this purpose (17). The intake of infrequently consumed nutrients (vitamin B_6_, vitamin B_12_, vitamin A retinol activity equivalent (RAE), vitamin B_3_, vitamin B_5_, and iodine) for each of the four recalls was considered for this study. The prevalence of inadequate intake of these nutrients was calculated using the probability approach (18) based on the dietary recommendations for Indian children (19).

### 2.4 Impact of zero inflation on estimation of habitual intake of infrequently consumed nutrient

A simulation study was carried out to assess the impact of varying proportions of zero inflation in observed intake on an estimated habitual intake of infrequently consumed nutrient. We assumed a scenario of *n* = 15 repeated recalls and generated a random sample (*Z*_*ij*_) of size 2,000 from a multivariate normal distribution with μ = (0.77, 0.74, 0.71, 0.61, 0.68, 0.78, 0.64, 0.67, 0.52, 0.61, 0.54, 0.61, 0.69, 0.78, 0.72) and Diag(Σ) = (1.70, 1.35, 1.41, 1.13, 0.91, 1.31, 1.29, 1.02, 1.50, 1.43, 1.34, 1.21, 1.13, 1.54, 1.27); σ_*ij*_ = ρσ_*i*_σ_*j*_. ρ = 0.6 was the within-individual correlation, Σ was the variance-covariance matrix, and Diag(Σ) was the diagonal element in the matrix, which corresponded to the variance. The variance-covariance matrix *Diag*(Σ) and the correlation coefficient ρ were obtained from the sample intake data of vitamin B_12_ for children as mentioned above, and the means were a range of values within the Recommended Dietary Allowance (RDA) for vitamin B_12_ in children and adolescents, which provided a reasonable range as the mean intake in the sample data was very low (less than the estimated average requirement (EAR) for the age group). Then, the actual intake was defined as *Y*_*ij*_ = *exp*(*Z*_*ij*_), which was a skewed distribution. Another random number from the binomial probability distribution with *n* = 15, *p* = {0.2, 0.3, …, 0.8} was generated for the positive intakes, where p was the proportion of positive intakes. To simulate zero inflation, a proportion (1-p) of the values of the series of 2,000 nutrient intakes *Y*_*ij*_ was replaced by 0. For each value of p, 2,000 samples of intakes were similarly generated. Then, both the ISUF model and MDM were fitted for each simulated dataset. The geometric mean and 95% confidence interval of estimated habitual intakes were computed (Supplementary Table 1). For comparison, habitual intake was also calculated as the arithmetic mean of 15 recalls, with results summarized as the geometric mean with a 95% confidence interval. Smoothened distribution curves of the habitual intakes estimated using the MDM, the ISUF method, and individual mean were plotted to visually assess the impact of varying proportions of zero intake.

## 3 Results

### 3.1 Estimation of habitual intake distribution of infrequently consumed nutrients

The data used for analysis consisted of 120 individuals with 4 recalls each. Vitamin B_6_, vitamin B_12_, vitamin B_3_, vitamin B_5_, vitamin A RAE, and iodine were considered for the application of this method, as they were consumed infrequently, with 17% non-consumers for vitamin B_12_, 9% non-consumers for vitamin B_6_ and B_5_, 2% non-consumers for vitamin A, 1% of non-consumers for vitamin B_3_, and 1% of non-consumers for iodine. Vitamins A and B_5_ and iodine were selected for the demonstration of the method, as they were positively skewed even though they were not inflated by zero intakes. The description of the observed nutrient intake from the example data is given in Table 1.

**Table 1:** Summary of observed nutrient intake in each recall (*n* = 120).

**Nutrient**		**First season**		**Second season**	
		**First recall**	**Second recall**	**First recall**	**Second recall**
Vitamin B_6_ mg	Mean ± SD	0.47 ± 0.37	0.49 ± 0.37	0.56 ± 0.33	0.54 ± 0.32
	Median (Q_1_, Q_3_)	0.39 (0.19, 0.65)	0.43 (0.22, 0.70)	0.50 (0.30, 0.79)	0.49 (0.30, 0.73)
Vitamin B_12_ μg	Mean ± SD	0.57 ± 0.64	0.57 ± 0.68	0.70 ± 0.79	0.67 ± 0.93
	Median (Q_1_, Q_3_)	0.43 (0.08, 0.86)	0.43 (0.05, 0.87)	0.54 (0.23, 0.91)	0.44 (0.17, 0.91)
Vitamin A mcg RAE	Mean ± SD	106 (77)	94 (68)	151 (89)	148 (93)
	Median (Q_1_, Q_3_)	92 (55, 138)	82 (50, 126)	134 (88, 196)	130 (78, 201)
Vitamin B_3_ mcg	Mean ± SD	4.9 (2.9)	5.0 (3.5)	4.6 (3.4)	4.5 (2.8)
	Median (Q_1_, Q_3_)	4.5 (2.7, 6.7)	4.2 (2.5, 6.7)	3.8 (2.2, 6.3)	4.2 (2.7, 5.9)
Vitamin B_5_ mg	Mean ± SD	0.99 (1.19)	0.95 (1.11)	0.81 (0.97)	0.94 (1.06)
	Median (Q_1_, Q_3_)	0.39 (0.09, 1.69)	0.40 (0.08, 1.43)	0.40 (0.07, 1.38)	0.52 (0.12, 1.49)
Iodine mcg	Mean ± SD	32 (27)	29 (20)	30 (25)	35 (30)
	Median (Q_1_, Q_3_)	25 (14, 43)	26 (13, 40)	26 (13, 43)	24 (16, 44)

SD, standard deviation; Q1, quartile 1; Q3, quartile 3; RAE, retinol activity equivalent.

**Figure 1:**
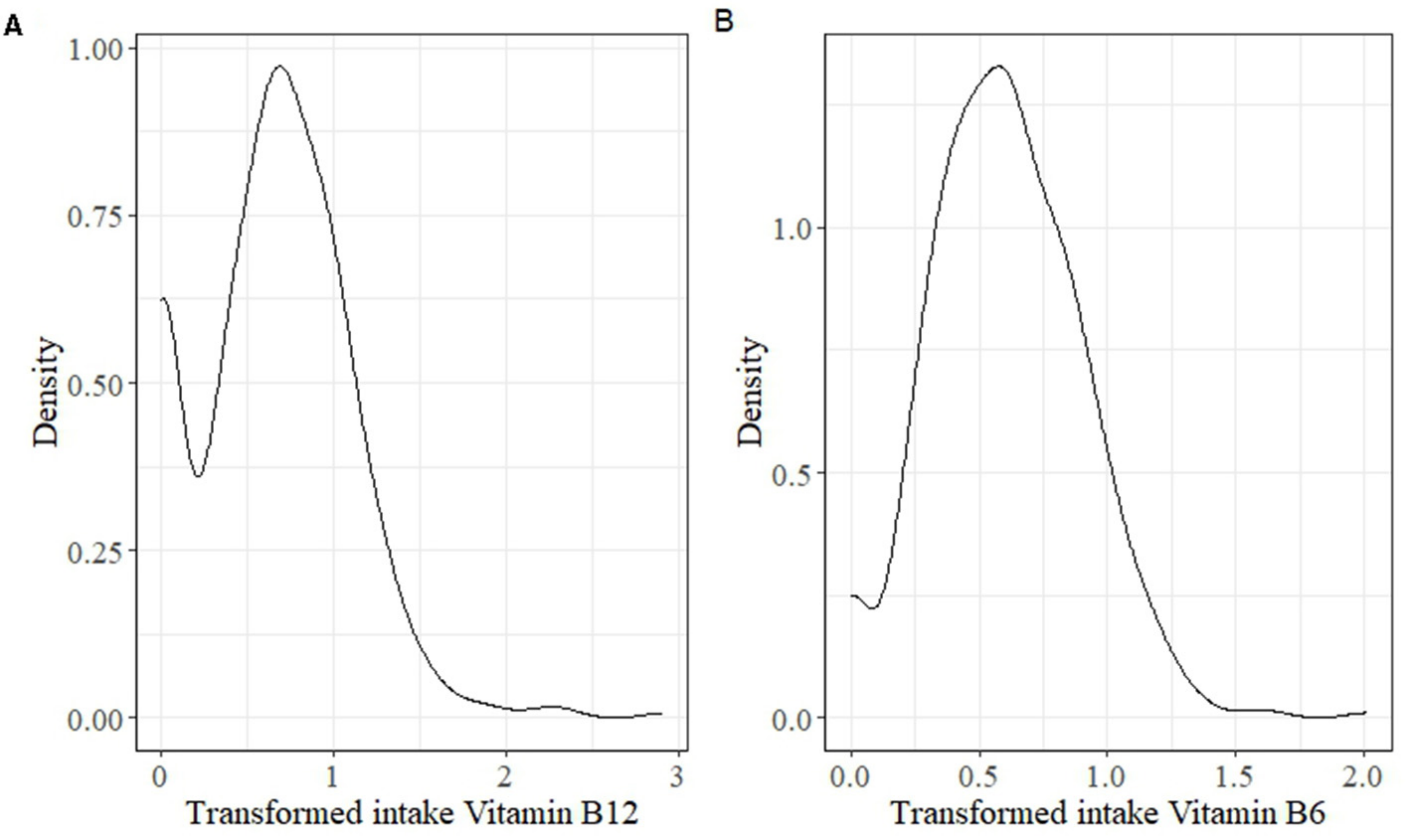
Density plot for examining the skewness of data after two-step transformation in the Iowa State University (ISU) method. **(A)** The transformed intake for vitamin B_12_, and **(B)** is the transformed intake for vitamin B_6_.

For the estimation of habitual intake of vitamins B_6_ and B_12_ from this sample, a two-step transformation was applied as suggested by the ISU method, where the first step was a power transformation, and then, a piecewise cubic estimate was performed as the second step. The transformed data was examined for the normality assumption. However, the density plot (Figure 1) of vitamins B_6_ and B_12_ showed that the transformed data were skewed and not meeting the requirement for the measurement error model used in the ISU method. Therefore, there is a need for a specialized method for estimating habitual intake distribution of infrequently consumed nutrients.

The ISUF method and the proposed MDM, which have been developed for infrequently consumed nutrients, were applied to the dietary intake of the nutrients under consideration. In the ISUF method, the habitual intake distribution of positive intakes was estimated using the ISU method. In the ISU method, the positive intakes of vitamins B_6_ B_3_, and A RAE were transformed to normality using power transformation. Powers of 0.3, 0.5, and 0.4 were sufficient to transform vitamins B_6_, B_3_, and vitamin A RAE to normal distribution, respectively. However, a two-step transformation was required for the positive intake of vitamin B_12_, vitamin B_5_, and iodine, which was highly skewed. The power used in the first step was 0.2 for vitamins B_12_ and B_5_ and 0.3 for iodine, and a piecewise cubic transformation, as explained in Nusser et al.'s (6) study, was used in the second step for these nutrients. The habitual intake of positive intakes in the transformed scale was estimated using the shrinkage estimator of measurement error model (5, 20).

Since the positive intake data of vitamins B_6_, B_3_, and A RAE were normally distributed after the power transformation, an inverse power transformation was used for converting positive habitual intake in the transformed scale to the original scale. For the more skewed distribution of positive intake of vitamin B_12_, vitamin B_5_, and iodine, which require two-step transformation, the relation between nutrient intake in the transformed scale and the original scale was developed using a polynomial curve fitting. The following relation was used for the back-transforming habitual intake of vitamin B_12_ in the transformed scale to the original scale.


$$\begin{array}{l} \text{Habitual vitamin B12 intake}=\;0.59\;-\;3.64\;\\ \;*\text{ Habitual vitamin B}12\text{ intake in normal scale }+\;8.52\\ \;*\;{\text{Habitual vitamin B}12\text{ intake in normal scale}}^{2}\;-\;9.47\\ \;*\;{\text{Habitual vitamin B}12\text{ intake in normal scale}}^{3}+\;5.0\\ \;*\;{\text{Habitual vitamin B}12\text{ intake in normal scale}}^{4} \end{array}$$

Thus, the estimation of habitual positive intake of nutrients was computationally intense in the ISUF method.

The goodness of fit of lognormal, gamma, and mixture normal distributions for the positive intakes of the nutrients was tested using the AIC (Supplementary Table 2). The AIC was lowest for gamma distribution for all nutrients except for vitamin B_6_ and closer to that of the lowest AIC of mixture normal distribution for vitamin B_6_. Thus, the gamma regression method was considered suitable for estimating habitual intake of positive intakes for all the nutrients under consideration.

In the MDM, the habitual intake distribution of positive intakes was directly estimated using the gamma regression method. Habitual intake was then estimated using a shrinkage estimator in the measurement error framework as given in Equation 4 (5, 20).

In the ISUF method, the probability of positive consumption was modeled as a discrete set of equally spaced probabilities (ranging from 0.0 to 1.0), and specific probability masses for 1, 2, 3, and 4 days of positive recalls were independently estimated.

The overdispersion parameter phi for the binomial distribution of the frequency of consumption was 2.2 for vitamins B_12_ and B_6_, 1.8 for vitamin A, 0.99 for vitamin B_3_ and iodine, and 1.2 for vitamin B_5_. Overdispersion was present in vitamins B_12_, B_6_, and A. The goodness of fit of binomial, Poisson, negative binomial, and beta-binomial distributions for the frequency of consumption of these nutrients was tested using the AIC (Supplementary Table 2). The AIC was lowest for the beta-binomial distribution for the nutrients except for vitamin B_3_ and iodine and for these two nutrients, the AIC was equal for the binomial and beta-binomial distributions. Hence, the beta-binomial regression method was considered suitable for estimating the probability of consumption for all these nutrients under consideration.

Thus, in the MDM, the frequency of consumption of the nutrients was modeled using beta-binomial distribution. Each individual's probability of consumption was estimated from the observed frequency of intake using the parameters of the beta-binomial distribution, as presented in Supplementary Table 3.

The distribution of habitual intake for each individual was then estimated as the product of estimated habitual intake on consumption days and the individual's probability of consumption. The descriptive statistics for estimated habitual intake are given in Table 2.

**Table 2:** Estimated habitual intake using different methods of estimation.

**Nutrient**	**Estimate using the ISUF method**		**Estimate using the MDM**	
	**Mean (**±**SD)**	**Median (Q**_1_ **, Q**_3_ **)**	**Mean (**±**SD)**	**Median (Q**_1_ **, Q**_3_ **)**
Vitamin B_6_ mg	0.48 (±0.26)	0.47 (0.29, 0.65)	0.46 (±0.26)	0.46 (0.29, 0.62)
Vitamin B_12_ μg	0.50 (±0.42)	0.40 (0.18, 0.69)	0.47 (±0.41)	0.38 (0.14, 0.68)
Vitamin A RAE μg	114 (±51)	113 (80, 141)	107 (±50)	107 (76, 134)
Vitamin B_3_ mg	4.21 (±1.08)	4.20 (3.69, 4.88)	4.02 (±1.26)	4.16 (3.48, 4.78)
Vitamin B_5_ mg	0.74 (±0.79)	0.26 (0.10, 1.23)	0.69 (±0.76)	0.24 (0.08, 1.21)
Iodine mg	25.56 (±8.36)	26.07 (20.76, 30.87)	25.0 (±9.96)	24.8 (19.4, 31.12)

ISUF, Iowa State University Foods; MDM, mixture distribution method; SD, standard deviation; Q1, quartile 1; Q3, quartile 3; RAE, retinol activity equivalent.

As shown in Table 2, it can be observed that the estimated habitual intakes using the MDM for vitamins B_6_ and B_12_ were comparable to the estimates using the complex ISUF method. The median and quartiles for vitamin B_6_ were 0.47 (0.29, 0.65) using the ISUF method and 0.46 (0.29, 0.62) using the MDM, although the method employed was much simpler and direct with no transformation of data. Similarly, comparable results were found for other nutrients as well. The habitual intake estimates stratified by age are given in Supplementary Table 4.

The prevalence of inadequacy was comparable for the habitual intakes estimated using the ISUF method and MDM (Table 3). The prevalence of inadequacy for habitual intake using the ISUF method and MDM was 93.6% (89.8%, 97.3%) and 95% (91.7%, 98.2%) for vitamin B_6_ and 89.1% (83.8%, 94.3%) and 89.6% (84.6%, 94.7%) for vitamin B_12_, respectively. The analysis showed comparable prevalence for inadequate intakes for other nutrients also. The prevalence of inadequate intake stratified by age is given in Supplementary Table 5.

**Table 3:** Prevalence of inadequate intake of the habitual intakes obtained using various methods.

**Nutrient**	**Prevalence of inadequacy (%)**	
	**ISUF method**	**MDM**
Vitamin B_6_ mg	93.6 (89.8, 97.3)	95.0 (91.7, 98.2)
Vitamin B_12_ mcg	89.1 (83.8, 94.3)	89.6 (84.6, 94.7)
Vitamin A RAE μg	55.7 (54.8, 56.7)	56.9 (55.5, 58.4)
Vitamin B_3_ mg	96.7 (94.5, 98.9)	96.7 (94.5, 98.9)
Iodine mg	99.5 (99.4 99.7)	99.4 (99.1, 99.7)

ISUF, Iowa State University Foods; MDM, mixture distribution method,

RAE, retinol activity equivalent.

### 3.2 Validation of the MDM using simulation

**Figure 2:**
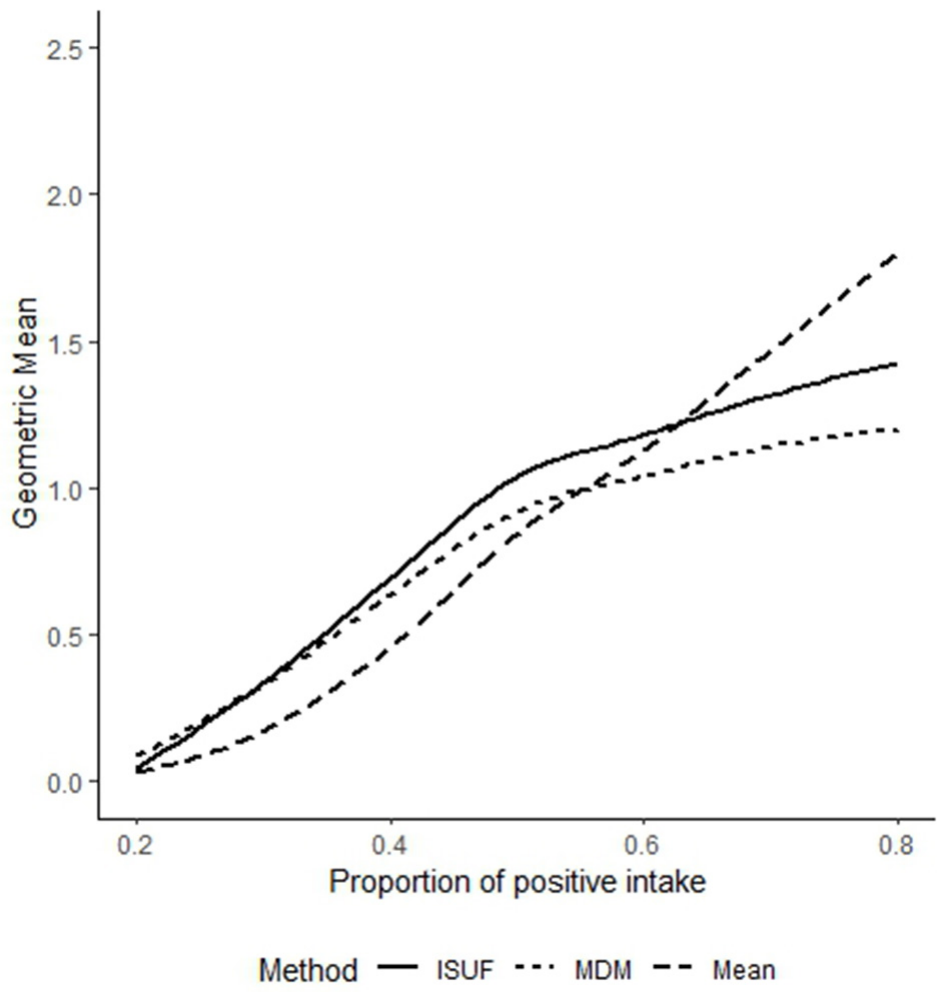
Line diagram for the geometric mean of estimated habitual intake distribution using the Iowa State University Foods (ISUF) method, mixture distribution method (MDM), and individual mean over 15 recalls for varying levels of positive consumption in the intake distribution using simulated data. The dashed line represents the estimated geometric mean of the individual mean, the dotted line represents the geometric mean of habitual intake estimated using the MDM, and the thick line represents the geometric mean of habitual intake estimated using the ISUF method.

Using the simulated data, the geometric mean of the estimated habitual intake distribution was obtained and plotted for visual comparison, as given in Figure 2.

Figure 2 illustrates that, when the proportion of positive intakes was below 60%, the habitual intakes estimated using the MDM and ISUF method were comparable and tended to exceed the individual mean. This was attributable to both methods incorporating the probability of zero intake, which helps mitigate the downward bias introduced by a high frequency of zero observations in the data. Thus, approaches that model the probability of non-consumption could yield more accurate estimates of habitual intake under zero-inflated conditions. Conversely, as the proportion of positive intakes exceeded 70%, the habitual intakes estimated by the MDM and ISUF method fell below the individual mean. This reflected the need for using models with the probability of positive consumption, such as the MDM and ISUF method, for estimating habitual intake of infrequently consumed nutrients.

## 4 Discussion

Accurate estimation of habitual intake is essential for developing evidence-based nutrition policies such as setting dietary reference values and designing fortification and supplementation programs (21). Estimation of habitual intake of nutrients is challenging when the intake data are skewed and become more complex for infrequently consumed nutrients due to zero inflation from non-consumption on the recall day. The proposed method, referred to as the MDM, addresses this using a two-part model that separately estimates the probability of consumption and the amount consumed on intake days. The probability of consumption was estimated by the modeling frequency of consumption as a beta-binomial distribution while intake amounts on consumption days were modeled using the gamma regression method. The simulation study by changing the proportion of positive intakes showed that the estimates using the MDM were closer to the individual means for lower proportions of positive intakes (< 60%) compared to the estimates using the ISUF method.

Studies have shown that the shape of habitual intake distributions varies by nutrient, country, gender, and age group, with vitamin intakes often displaying greater variability (22). These differences influence the estimated prevalence of inadequate intake. Understanding intake distributions and applying appropriate methods to estimate habitual intake directly impact the assessment of inadequacy and the evaluation of nutrition interventions. Studies have modeled skewed intake data using the gamma distribution to estimate inadequacy (23) or summarized intake without normal transformation or measurement error correction (15). Nutrient intake was modeled using gamma distribution in studies where the adjustment on the variability was performed using the variance ratio from external sources and applied on the parameters of the observed intake distribution (24). Then the gamma distribution, adjusted for the external variance ratio was used to represent the habitual intake distribution.

Modeling the consumption frequency using beta-binomial distribution was suggested in previous studies (13, 25). However, the estimation of habitual intake based on the amount of consumption was performed by transforming the data to normal distribution. The Statistical Program to Assess Dietary Exposure (SPADE) also uses beta-binomial distribution for estimating the probability of consumption. However, the SPADE method requires the positive intake to follow normal distribution. The amount of consumption was modeled using a linear mixed effect model after transforming to normal distribution using Box–Cox transformation and back-transforming using Gaussian quadrature (10). The estimation using MSM (11) handled zero-inflated dietary data by distinguishing between consumers and non-consumers, thereby integrating data from 24-h recall and food frequency questionnaire (FFQ). In this method, the probability of consumption was estimated using a logistic regression model for consumers and not by recall, and the habitual intake was estimated after transforming data with the help of Box–Cox transformation. This method cannot be preferred while dealing with highly skewed data. One of the limitations of this method lies in the transformation to normal distribution and back-transformation process, which may introduce bias if not handled appropriately. The advantage of the MDM method proposed in this study is that it models intake data based on the actual distribution of positive intake using the gamma regression method.

The method developed by the National Cancer Institute, known as the NCI method, also uses the two-part model where the probability of consumption was predicted using the logistic model, and the amount consumed was transformed to normality using the Box–Cox transformation (7). It incorporated individual and recall-specific covariates into the model. This procedure of estimation of the parameters is complicated. The use of the MDM reduces the complexity in estimating habitual intake.

An ensemble approach had been proposed for estimating habitual intake from a single 24-h recall where the variance ratio could be obtained from an external source (8). However, the usage of the external variance ratio of between- and within-variability does not address the problem of skewed and infrequent intake of nutrients.

The limitation of the existing methods of estimating infrequently consumed nutrients was that the transformation to normal and back transformation was complex due to high skewness in the consumption data of infrequently consumed nutrients. Such transformations, if not handled appropriately, can introduce bias to the estimates. The advantage of the MDM method proposed in this study addresses these challenges by directly modeling the actual distribution of the positive intake using the gamma regression method and the probability of intake using the beta-binomial regression method.

Studies discussing the minimum sample size required for the estimation of habitual intake state that at least 50 individuals with at least 2 recalls are sufficient to estimate habitual intake using a measurement error model (4, 26). This holds good for nutrients consumed regularly, as the multiple recalls will be capturing the consumption of that nutrient. However, for infrequently consumed nutrients, the estimates might be biased if the probability of consumption of the nutrient on any given day is low. Differentiating between occasional consumers and true non-consumers is not possible with a limited number of recalls. Therefore, either the sample size or the number of repeated recalls needs to be higher to ensure a sufficient number of individuals with at least two positive intakes are presented in the data. Sample size can affect the estimated habitual intake, as the bias reduces with increased sample size (27). The dietary data of 120 children with 4 recalls each, used to demonstrate the proposed MDM, can be considered sufficient for the estimation of habitual intake, as the estimates were comparable with the ISUF method (4, 13).

The application of the MDM method to larger and diverse intake data is needed to examine the effects of sample size and the minimum number of recalls required for estimating habitual intake of infrequently consumed nutrients.

The MDM needs to be explored further to accommodate factors associated with positive consumption. Additionally, the incorporation of weights for varying numbers of recalls across participants in a study also needs to be investigated to improve generalizability. While the current study focuses on nutrient intake, the MDM could be extended to estimate habitual intake of infrequently consumed foods. However, such an extension must carefully distinguish between true non-consumers and infrequent consumers to ensure accurate estimation.

## 5 Conclusion

There are several challenges in estimating the habitual intake of infrequently consumed nutrients when the number of repeated 24-h recalls available is low. This study proposes a computationally simpler MDM that modeled the frequency of consumption of the nutrient and the amount consumed. The suggested MDM method is straight forward and helps to estimate habitual intake for infrequently consumed nutrients precisely and accurately for zero-inflated infrequently consumed nutrient data.

## Data Availability

Data described in the manuscript will be made available upon request. Requests to access these datasets should be directed to Dr. Tinku Thomas, tinku.sarah@sjri.res.in.
