# Supplementary material for: Estimation of habitual intake of infrequently consumed nutrients using the mixture distribution method
Source: Front Nutr. 2025 Nov 10;12:1631495. doi: 10.3389/fnut.2025.1631495 (PMC12641393; doi:10.3389/fnut.2025.1631495)
Supplement: Supplementary file 1 [file Table_1.docx]

Supplementary Table 1: Geometric mean of the habitual intakes estimated using different methods

| Proportion of positive intakes | **Geometric Mean (95% CI) of habitual intake** | | |
| --- | --- | --- | --- |
|  | Individual mean | ISUF method | MDM |
| 0.8 | 1.80 (1.72,1.88) | 1.43 (1.36, 1.49) | 1.20 (1.15, 1.25) |
| 0.7 | 1.48 (1.40, 1.56) | 1.32 (1.28, 1.37) | 1.15 (1.10, 1,20) |
| 0.6 | 1.13 (1.05, 1.21) | 1.18 (1.16, 1.20) | 1.04 (0.99, 1.09) |
| 0.5 | 0.85 (0.75, 0.95) | 1.04 (0.98, 1.10) | 0.92 (0.86, 0.98) |
| 0.4 | 0.46 (0.38, 0.54) | 0.69 (0.64, 0.75) | 0.64 (0.59, 0.69) |
| 0.3 | 0.17 (0.10, 0.24) | 0.32 (0.28, 0.36) | 0.31 (0.26, 0.36) |
| 0.2 | 0.03 (0.01, 0.07) | 0.05 (0.03, 0.07) | 0.09 (0.06, 0.12) |

ISUF: Iowa State University Foods method, MDM: Mixture Distribution Method

Supplementary Table 2: Akaike Information Criterion (AIC) for the fitted distribution for positive intake and for the frequency of consumption

| **AIC for the fitted distribution for positive intake** | | | | |
| --- | --- | --- | --- | --- |
| **Nutrient** | **Normal distribution** | **Lognormal distribution** | **Gamma distribution** | **Mixture Normal distribution** |
| Vitamin B_12_ µg | 188.86 | 130.79 | 122.67 | 134.78 |
| Vitamin B_6_ mg | 41.37 | 56.06 | 35.27 | 29.74 |
| Vitamin A RAE µg | 5509.79 | 5433.22 | 5371.08 | 5398.79 |
| Vitamin B_3_ mg | 2449.63 | 2477.15 | 2347.25 | 2445.63 |
| Vitamin B_5_ mg | 1320.4 | 893.10 | 858.47 | 934.28 |
| Iodine mg | 4439.20 | 4276.47 | 4188.08 | 4261.03 |
| **AIC for the fitted distribution for frequency of consumption** | | | | |
| **Nutrient** | **Binomial distribution** | **Poisson distribution** | **Negative binomial distribution** | **Beta-binomial distribution** |
| Vitamin B_12_ µg | 325.33 | 422.67 | 424.67 | 260.05 |
| Vitamin B_6_ mg | 146.06 | 402.44 | 404.44 | 109.32 |
| Vitamin A RAE µg | 79.00 | 396.22 | 396.22 | 67.32 |
| Vitamin B_3_ mg | 30.11 | 393.87 | 393.87 | 30.11 |
| Vitamin B_5_ mg | 191.44 | 395.14 | 395.14 | 189.96 |
| Iodine mg | 43.72 | 393.84 | 393.84 | 43.72 |

Supplementary Table 3: Parameters used for estimation in mixture distribution method (MDM)

| **Nutrient** | **Variability of positive intakes obtained using gamma regression** | | **Estimates of probability mass in MDM method** | | | | |
| --- | --- | --- | --- | --- | --- | --- | --- |
|  | Within individual variability ($\hat{\sigma}_{u})$ | Between individual variability ($\hat{\sigma}_{y})$ | p_0_ | p_1_ | p_2_ | p_3_ | p_4_ |
| Vitamin B_6_ mg | 0.45 | 0.44 | 0.008 | 0.023 | 0.048 | 0.100 | 1 |
| Vitamin B_12_ µg | 0.63 | 0.58 | 0.04 | 0.1 | 0.189 | 0.342 | 1 |
| Vitamin A RAE µg | 0.29 | 0.16 |  | 0.01 | 0.02 | 0.06 | 1 |
| Vitamin B_3_ mg | 0.39 | 0.05 |  |  |  | 0.02 | 1 |
| Vitamin B_5_ mg | 0.54 | 0.88 |  | 0.01 | 0.06 | 0.30 | 1 |
| Iodine mg | 0.51 | 0.11 |  |  |  | 0.04 | 1 |

Probability mass (p_i_) was estimated using beta-binomial regression using the frequency of consumption out of 4 recall days (i=0,1,2,3,4)

Supplementary Table 4: Age-wise habitual intake of nutrients using different methods

| **Nutrient** | **Estimate using ISUF method** | | | | | | **Estimate using MDM method** | | | | | |
| --- | --- | --- | --- | --- | --- | --- | --- | --- | --- | --- | --- | --- |
|  | **< 12 months** | | **13-36 months** | | **> 36 months** | | **< 12 months** | | **13-36 months** | | **> 36 months** | |
|  | Mean (SD) | Median (Q_1_, Q_3_) | Mean (SD) | Median (Q_1_, Q_3_) | Mean (SD) | Median (Q_1_, Q_3_) | Mean (SD) | Median (Q_1_, Q_3_) | Mean (SD) | Median (Q_1_, Q_3_) | Mean (SD) | Median (Q_1_, Q_3_) |
| Vitamin B_6_ mg | 0.20 (0.15) | 0.18 (0.07, 0.30) | 0.45 (0.18) | 0.43 (0.31, 0.56) | 0.76 (0.18) | 0.74 (0.60, 0.89) | 0.17 (0.16) | 0.17 (0.01, 0.30) | 0.43 (0.17) | 0.42 (0.31, 0.54) | 0.73 (0.17) | 0.72 (0.59, 0.84) |
| Vitamin B_12_ µg | 0.47 (0.55) | 0.24 (0.03, 0.82) | 0.50 (0.40) | 0.39 (0.19, 0.68) | 0.51 (0.35) | 0.43 (0.29, 0.69) | 0.45 (0.54) | 0.24 (0.02, 0.81) | 0.47 (0.40) | 0.38 (0.16, 0.67) | 0.48 (0.33) | 0.41 (0.22, 0.68) |
| Vitamin A mcg RAE | 77 (57) | 59 (30, 126) | 110 (38) | 110 (81, 129) | 148 (46) | 142 (108, 170) | 68 (57) | 54 (4, 125) | 105 (36) | 104 (76, 127) | 141 (45) | 137 (106, 161) |
| Vitamin B_3_ mcg | 4.33 (1.29) | 4.16 (3.74, 4.94) | 4.27 (0.98) | 4.35 (3.70, 4.88) | 4.00 (1.09) | 4.16 (3.51, 4.51) | 4.19 (1.44) | 4.14 (3.65, 4.85) | 4.07 (1.15) | 4.27 (3.50, 4.77) | 3.80 (1.33) | 4.01 (3.42, 4.59) |
| Vitamin B_5_ mg | 0.68 (0.78) | 0.23 (0.10, 1.26) | 0.68 (0.78) | 0.19 (0.07, 1.18) | 0.91 (0.81) | 0.69 (0.15, 1.57) | 0.64 (0.76) | 0.23 (0.07, 1.21) | 0.63 (0.75) | 0.18 (0.05, 1.09) | 0.86 (0.79) | 0.60 (0.14, 1.48) |
| Iodine mcg | 27 (8) | 29 (22, 31) | 26 (9) | 26 (19, 31) | 24 (8) | 24 (22, 30) | 26 (10) | 29 (20, 32) | 25 (10) | 25 (18, 31) | 24 (10) | 24 (19, 30) |

ISUF: Iowa State University Foods, MDM: Mixture Distribution Method, SD: standard deviation, Q1: quartile 1, Q3: quartile 3, RAE: retinol activity equivalent

Supplementary Table 5: The prevalence of inadequate intake of the habitual intakes obtained using various methods stratified by age

| **Nutrient** | **Prevalence of inadequacy (%) (95% CI)** | | | | | |
| --- | --- | --- | --- | --- | --- | --- |
|  | **6-12 months (n=25)** | | **13-36 months (n=65)** | | **> 36 months (n=33)** | |
|  | **ISUF** | **MDM** | **ISUF** | **MDM** | **ISUF** | **MDM** |
| Vitamin B_6_ mg | 95.9 (88.2, 100) | 96.2 (88.8, 100) | 96.7 (90.0, 100) | 97.2 (91.2, 100) | 85.9 (75.3, 96.5) | 89.9 (81.6, 98.2) |
| Vitamin B_12_ mcg | 83.3 (69.2, 97.3) | 84.5 (71.3, 97.7) | 85.6 (77.3, 93.8) | 86.2 (78.2, 94.2) | 100 (100, 100) | 100 (100, 100) |
| Vitamin A mcg RAE | 60.3 (56.7, 63.9) | 64.6 (59.0, 70.2) | 54.7 (53.9, 55.5) | 55.1 (54.3, 55.9) | 54.3 (53.4, 55.2) | 54.7 (53.8, 55.6) |
| Vitamin B_3_ mcg |  |  | 94.9 (91.1, 98.7) | 94.9 (91.1, 98.7) | 100 (100, 100) | 100 (100, 100) |
| Iodine mcg |  |  | 99.5 (99.2, 99.8) | 99.2 (98.7, 99.7) | 99.7 (99.5, 100) | 99.6 (99.2, 100) |

ISUF: Iowa State University Foods, MDM: Mixture Distribution Method, CI: confidence interval

Dietary recommendations were not available for vitamin B_5_ for among age groups and for vitamin B3 and iodine among <12 months children

**Supplementary material 1: R code for estimation of habitual intake of infrequently consumed nutrients**

library(MASS)

library(lme4)

library(VGAM)

#Data consists of two variables 'ID' and nutrient intake named as 'Intake' with 4 recalls each, entered in long format

#Estimation of positive intake using Gamma regression

#removing zero intakes from data

DataP=subset(Data,Data$Intake>0)

1r=4 #number of recalls

#Fitting gamma regression on positive intakes

fit=glmer(Intake~(1|ID),data=DataP,family = Gamma(link=log));summary(fit)

Mu=fixef(fit)

s<-as.data.frame(VarCorr(fit))

y<-tapply(log(DataP$Intake),DataP$ID,mean)

HIP<-exp(Mu+sqrt(s[2,"vcov"]/(s[2,"vcov"]+s[1,"vcov"]/r))*(y-Mu)) ##habitual intake of positive intakes

#obtaining the frequency of consumption from observed intake

Data$freq=ifelse(Data$Intake==0,0,1)

Freq=tapply(Data$freq,Data$ID,sum)

#Probbaility of consumption using beta binomial distribution

P=pbetabinom.ab(Freq,r, shape1 = Coef(vglm(cbind(Freq,r-Freq)~1,betabinomialff))[1], shape2 = Coef(vglm(cbind(Freq,r-Freq)~1,betabinomialff))[2], log = FALSE)

Dat=data.frame(Freq,P)

Freq1=subset(Dat,Dat$Freq>0)

Freq0=subset(Dat,Dat$Freq==0)

FreqA=rbind(Freq0,Freq1)

#=========================================

HI=c(rep(mean(exp(y))/1000,nrow(Freq0)),HIP)*FreqA$P ##habitual intake of consumption in any given day
