# Supplementary material for: Estimation of habitual intake of infrequently consumed nutrients using the mixture distribution method
Source: Front Nutr. 2025 Nov 10;12:1631495. doi: 10.3389/fnut.2025.1631495 (PMC12641393; doi:10.3389/fnut.2025.1631495)

Estimation of Habitual Intake of Infrequently Consumed Nutrients Using Mixture Distribution Method

First author: Smitha Joseph

Supplementary Figure 1

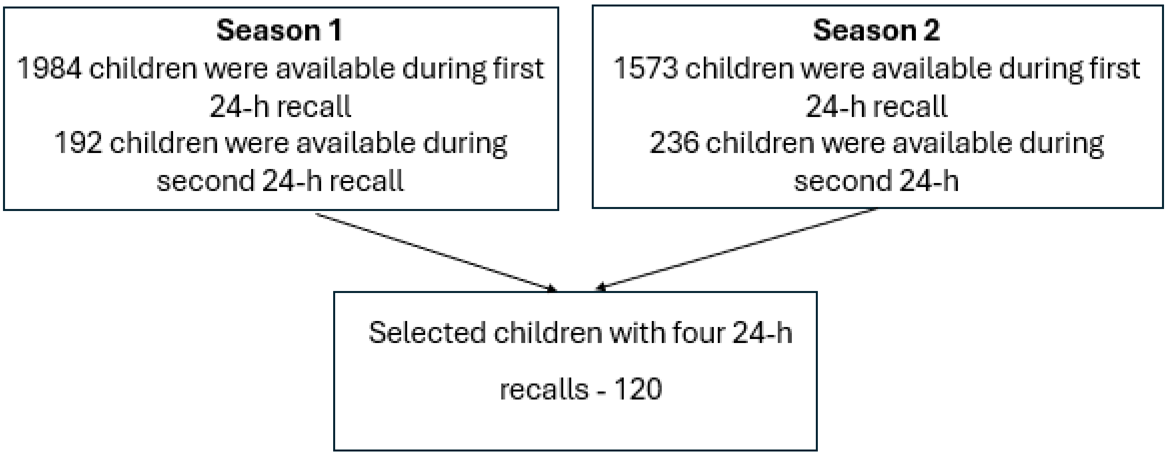

Supplement: Supplementary file 2 [file Image_1.pdf]
